# A pilot study of the quality of care of atrial fibrillation in Irish general practice

**DOI:** 10.1093/fampra/cmae001

**Published:** 2024-01-30

**Authors:** Sarah McErlean, John Broughan, Geoff McCombe, Ronan Fawsitt, Mark Ledwidge, Walter Cullen, Joe Gallagher

**Affiliations:** Department of General Practice, UCD School of Medicine, University College Dublin, Belfield, Dublin 4, Ireland; The Palms General Practice Surgery, The Avenue, Gorey, Co. Wexford, Ireland; Department of General Practice, UCD School of Medicine, University College Dublin, Belfield, Dublin 4, Ireland; Department of General Practice, UCD School of Medicine, University College Dublin, Belfield, Dublin 4, Ireland; Department of General Practice, UCD School of Medicine, University College Dublin, Belfield, Dublin 4, Ireland; The Surgery Castle Gardens Kilkenny Co, Kilkenny, Ireland; Department of General Practice, UCD School of Medicine, University College Dublin, Belfield, Dublin 4, Ireland; Department of General Practice, UCD School of Medicine, University College Dublin, Belfield, Dublin 4, Ireland; Summerhill Family Practice, Summferhill, Dublin 1, Ireland; Department of General Practice, UCD School of Medicine, University College Dublin, Belfield, Dublin 4, Ireland; The Palms General Practice Surgery, The Avenue, Gorey, Co. Wexford, Ireland

**Keywords:** atrial fibrillation, general practice, guideline adherence, Ireland, observational study, quality of health care

## Abstract

**Background:**

Worldwide, atrial fibrillation (AF) is the most common sustained cardiac arrhythmia in adults and poses a significant burden to patients, physicians, and healthcare systems. We developed a quality of care score based on the Atrial Fibrillation Better Care pathway recommended by the European Society of Cardiology and the European Heart Rhythm Association guidelines. This is a 14-point score that we have termed the MAGIC score(Management of Atrial Fibrillation in Integrated Care and General Practice).

**Objective:**

The objective of this pilot study was to develop and test a quality of care score for patients with permanent AF in general practice.

**Methods:**

An observational cross-sectional pilot study was undertaken. Proportionate sampling was used across 11 practices from the Ireland East practice-based research network. The GPs completed a report form on each patient by undertaking a retrospective chart review. Eleven practices participated with a total of 1855 patients with AF. We received data on 153 patients.

**Results:**

The main findings were that no patient met all 14 guideline based recommendations. The mean MAGIC score was 11.3. Points were most commonly deducted because the creatinine clearance and HAS-BLED score were not recorded, and the patient was not on the correct dose of oral anti-coagulation.

**Conclusion:**

This study demonstrates the feasibility of using a quality of care score to measure the quality of AF management in general practice. This scoring system, which is based on internationally recognized quality of care markers, highlights key areas that can be targeted with quality improvement intervention.

**Key messages**

- Atrial fibrillation is the most common arrythmia worldwide
- Using a quality of care score is a feasible way to measure quality
- The HAS-BLED score and Creatine Clearance are often not recorded
- These measures could be automated in the general practice software system

## Introduction

Atrial fibrillation is the most common sustained cardiac arrhythmia in adults across the world and poses a significant burden to patients, physicians, and healthcare systems globally.^1^ The prevalence of AF is rising due to an ageing population, more vigorous searching for undiagnosed AF, and the increasing burden of co-morbidities.

Despite progress in the management of AF, it is still a leading cause of stroke, heart failure, and cardiovascular morbidity and mortality.^2^ AF is also associated with reduced quality of life (QoL), depression, cognitive decline, and hospitalization.^2,3^ Adherence to guideline-directed therapy can improve outcomes in AF, and yet there are wide variations in compliance to these guidelines.^4–8^

The latest ESC guidelines highlight that patients with AF have a broad range of adverse cardiovascular outcomes, and the approach to management should be comprehensive and holistic. This can be achieved using the Atrial Fibrillation Better Care (ABC) pathway.^1^ This ABC pathway streamlines integrated care of AF patients across all levels and specialities of healthcare and has shown significant benefits and improved clinical outcomes.^1,9–11^

Given the prevalence of AF, optimal management is paramount to improve quality of life and reduce the overall impact of AF on the health and social care system.^12^ Measuring quality and identifying opportunities for improvement is an essential part of optimal AF management.^1^ Guidelines are rarely absorbed organically into clinical practice sufficiently enough to create a meaningful change. Additional QI interventions are often needed, and usually a combination of education, decision support, ongoing audit and feedback, and organizational change are necessary for a sustained effect.^13^ Good quality data acquisition, audit, and feedback are essential to highlight areas in need of intervention and monitor progress. We know that audit and feedback can lead to important improvements in clinical care and drive behaviour change.^14,15^

The aim of this pilot study is to develop and test the feasibility of using a quality of care score called the MAGIC score (Management of Atrial Fibrillation in Integrated Care and General Practice) for patients with permanent atrial fibrillation in general practice.

## Methods

### Development of quality of care score

Quality indicators (QIs) serve both as a tool to assess the quality of care delivery and as an instrument to judge the effectiveness of quality improvement initiatives. Measuring QIs enables health care professionals (HCPs) to determine, compare, and improve adherence to guidelines.

The European Heart Rhythm Association (EHRA) developed a set of QIs that can be used to evaluate the quality of care and outcomes for adults with atrial fibrillation.^2^ In total, EHRA developed 17 main and 17 secondary outcomes in 6 domains of care for the diagnosis and management of AF.

Our MAGIC score is derived from these QIs. The score was developed by an expert panel of general practitioners using EHRA criteria that were (i) relevant to general practice, (ii) extractable from the patient record, and (3) amenable to a quality improvement intervention.

We have included all main quality indicators from Domain 1: Patient assessment, Domain 2: Anticoagulation, Domain 3: Rate control, and Domain 5: Risk factor management. We have not included any QIs from Domain 4: Rhythm Control as the rhythm control strategy for AF is managed in secondary care in Ireland. We have not included Domain 6: Outcomes, as this was a cross-sectional observation study, and we did not assess patient outcomes. Table 1 maps the MAGIC score to the EHRA QIs.

**Table 1.** MAGIC* Score for quality of care in general practice mapped to EHRA Quality indicators for the care of adults with atrial fibrillation.

		EHRA Quality Indicator^2^
		Domain 01: Patient assessment
1	CHA_2_DS_2_-VASc score recorded since diagnosis	*01MQI1: Proportion of patients with cardio-embolic risk assessment using CHA*_*2*_ *DS*_*2*_ *-VASc score.* To assess the risk of stroke, a risk factor-based approach is recommended using the CHA_2_DS_2_-VASc clinical stroke risk score in all patients with AF.^1,2^
2	HAS-BLED score recorded since diagnosis	*01MQI2: Proportion of patients with bleeding risk assessment using a validated method, such as the HAS-BLED score.* A bleeding risk assessment is used to identify those at higher risk of bleeding who may need closer follow-up and to focus attention on modifiable bleeding risk factor.^1^ Using the HAS-BLED score can reduce bleeding risk and increase OAC use.^16^
3	Renal function measured since diagnosis	*01MQI3 Proportion of patients with a measurement of their serum creatinine (or creatinine clearance)* Measuring renal function has implications for stroke risk, bleeding risk and dose of OAC.^17^ Calculation of creatinine clearance is required for appropriate NOAC dosing.^17^
4	Creatinine Clearance calculated since diagnosis	*01MQI3 Proportion of patients with a measurement of their serum creatinine (or creatinine clearance)*
		Domain 02: Anticoagulation
5	Appropriately prescribed anticoagulation according to CHA_2_DS_2_-VASc score	*02MQI1: Proportion of patients who are appropriately prescribed anticoagulation according to CHA*_*2*_ *DS*_*2*_ *-VASc score* *02MQI2:Proportion of patients with a CHA*_*2*_ *DS*_*2*_ *-VASc score of 0 for men and 1 for women who are inappropriately prescribed long-term anticoagulation* The ESC 2020 guidelines recommend OAC for stroke prevention in males ≥ 1 and in females with a score of ≥ 2.^1^ This domain will assess the number of patients appropriately anti-coagulated and those at low risk who are inappropriately anti-coagulated.
6	Prescribed correct NOAC dose	02MQI3: Proportion of patients with ‘appropriate anticoagulation’ defined as: Appropriate dose for NOAC according to manufacturer recommendations. Under and over dosing of NOAC therapy is common and associated with worse outcomes.^18,19^
		Domain 03: Rate control
7	Patients with permanent AF who are not inappropriately prescribed antiarrhythmic drugs	*03MQI1: Proportion of patients with permanent AF (i.e. where no attempt to restore sinus rhythm is planned), who are inappropriately prescribed antiarrhythmic drugs.* Antiarrhythmic drugs e.g. amiodarone are not recommended when no further attempts to restore sinus rhythm are planned.^1,2^
		Domain 05: Risk factor management Comprehensive risk factor management complements stroke prevention, reduces AF burden, symptom severity and recurrence.^1^ A large proportion of these risk factors are lifestyle related and therefore amenable to modification, e.g. hypertension,^1^ diabetes,^20^ weight.^21^
8	TSH evaluation since diagnosis	05MQI1: Proportion of patients who have their modifiable risk factors identified
9	Fasting glucose/ HBa1C measurement since diagnosis	05MQI1: Proportion of patients who have their modifiable risk factors identified
10	Echocardiography performed since diagnosis	05MQI1: Proportion of patients who have their modifiable risk factors identified
11	BP measurement since diagnosis	05MQI1: Proportion of patients who have their modifiable risk factors identified
12	BP < 140/90 mmHg	05MQI1: Proportion of patients who have their modifiable risk factors identified
13	On anti-platelet only if indicated	05MQI1: Proportion of patients who have their modifiable risk factors identified
14	Weight recorded	05MQI1: Proportion of patients who have their modifiable risk factors identified

^*^MAGIC = Management of Atrial fibrillation in General practice and Integrated Care

### Study design

A descriptive, cross-sectional observational study was undertaken. This was a pilot study to determine the feasibility of using the MAGIC quality of care score.

### Setting

The study involved general practices (*n* = 14) participating in a research network on the east coast of Ireland. GPs who agreed to participate (*n* = 11) returned a signed consent form and the total number of patients with permanent AF in their practice. Proportionate sampling was used to generate a sample size for individual practices.

A random number generator was used to identify patients from their individual lists in each practice to include in the study. A paper case report form was used to collect defined anonymised data on these patients. The report forms were returned to the research team in UCD. These data were then cleaned, pooled, and inputted onto a secure database.

### Participants

All practices in the Ireland East practice-based research network were invited to participate.

They identified patients in the practice with permanent AF.

#### Inclusion criteria

Age ≥ 18 years

Permanent AF detected on ECG, Holter recording, or event recorder

Active in practice software and has attended the practice in the last 24 months

#### Exclusion criteria

No electrocardiographic objectified AF

### Study size

As this was a pilot study a formal sample size was not calculated.^22^ Based on previous work, it was estimated that 12 per group would be sufficient.^23,24^ We estimated that 80% of the practice would complete the study (11 practices). Therefore, we sought to recruit a minimum of 132 patients using proportional sampling.

### MAGIC score

Each participant file was reviewed using the MAGIC score, and points were awarded if the relevant component was present. Points were not awarded if the information was not recorded or could not be determined. Each component of the score was equally weighted with a maximum score of 14.

### Statistical methods

RedCap^25^ was used for data storage and analysis. The SPSS programme was used for statistical analysis. Missing data were either excluded from analysis or calculated based on information provided, e.g. creatinine clearance.

## Results

Eleven practices consented to participate in this pilot study with a total number of 1,855 patients with AF. We received data on 153 patients proportionally sampled across these 11 practices.

The demographic details can be seen in Table 2.

**Table 2.** 

Patient Demographics							
Gender	Male	Female	Missing				
	104	46	3				
Age	Min	Max	Mean	Median	Missing		
	48 years	96 years	76 years	78 years	3		
Medical Card Status	GMS	DVC	Private	Missing			
	116	11	17	9			
CDM Review	Yes	No	Missing				
	115	37	1				
Smoking Status	Current	Ex-smoker	Never smoker	Missing			
	17	52	75	9			
Alcohol Intake (units/ week)	Min	Max	Mean	Missing			
	0	140	4.8	24			
BMI	Min	Max	Mean	Median	Missing		
	17	45	29.2	28.4	30		
Co-Morbidities	Hypertension	CAD	Diabetes	Stroke/ TIA	PVD	OSA	Hyperthyroid
	79	57	41	23	7	6	3

CAD, coronary artery disease; DVC, doctor visit card; GMS, general medical services; OSA, obstructive sleep apnoea; PVD, peripheral vascular disease; TIA, transient ischaemic attack.

Most patients—68% were male (*N* = 104), and the mean age was 76 years. The majority of patients (83%) had a “General Medical Service” (GMS) card or Doctor Visit Card (DVC), which entitles patients to free GP care in Ireland. These are awarded to all patients over 70 years, under 6 years of age, and those below a certain income level based on means testing. These patients are also eligible for a biannual structured chronic disease management (CDM) review which was introduced in March 2020. This CDM review is not available to patients without a GMS or DVC card. Most patients had a CHA_2_DS_2_-VASc score calculated (76%) and most patients (86%) were on oral anticoagulation (OAC). The most commonly used OAC was Apixaban (52.3%). Of the 21 patients not on OAC, this was only appropriate in 4 of them based on their CHA_2_DS_2_-VASc score.

**Fig. 1.**
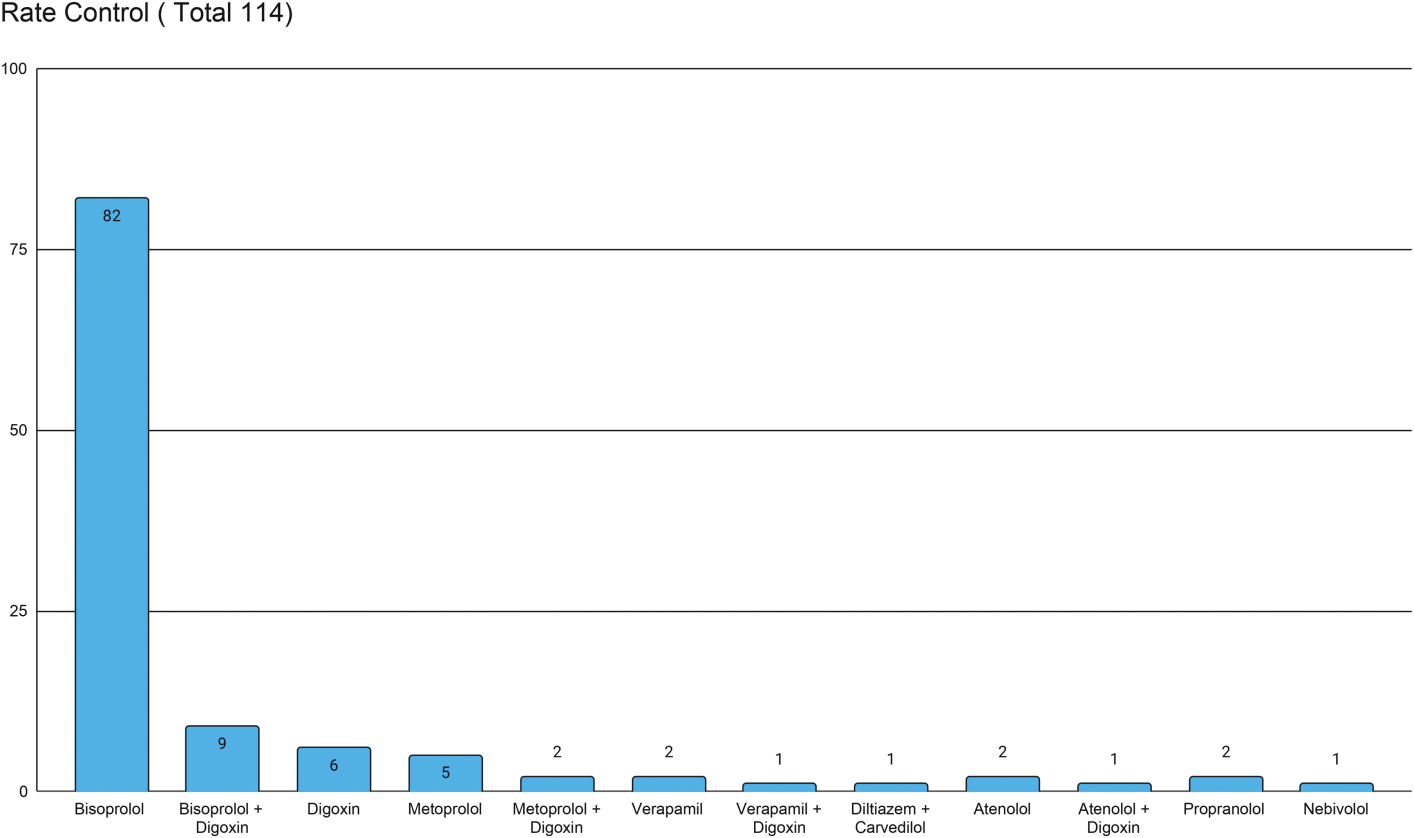
Rate control. Rate control medication

The mean heart rate was 74 bpm (rang 42–124 bpm), and the mean blood pressure was 132/77 mmHg (systolic BP range 90–207 mmHg, diastolic BP range 51–103 mmHg). 75% of patients were on rate-controlling medication. 99.3% of patients had a HR < 110 bpm. Bisoprolol was the most common medication used (54%) (Fig. 1). 12% of patients were prescribed anti-arrhythmic drugs despite being in permanent atrial fibrillation.

The mean BMI was 29.2 kg/m^2^ (range 17–45 kg/m^2^) with 36.4% being above 30 kg/m^2^. 45% were current or ex-smokers, 49% never smoked, and the mean weekly alcohol intake was 4.8 units. Hypertension was the most common co-morbidity recorded followed by coronary artery disease.

**Fig. 2.**
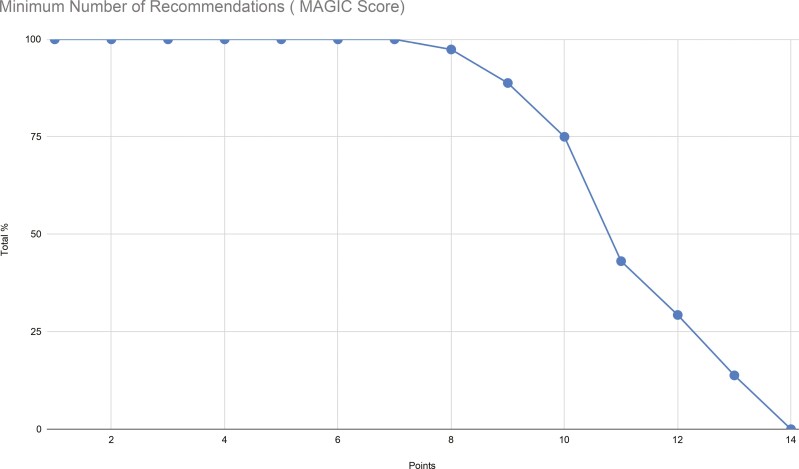
Minimum no. of recommendations (MAGIC score).

**Fig. 3.**
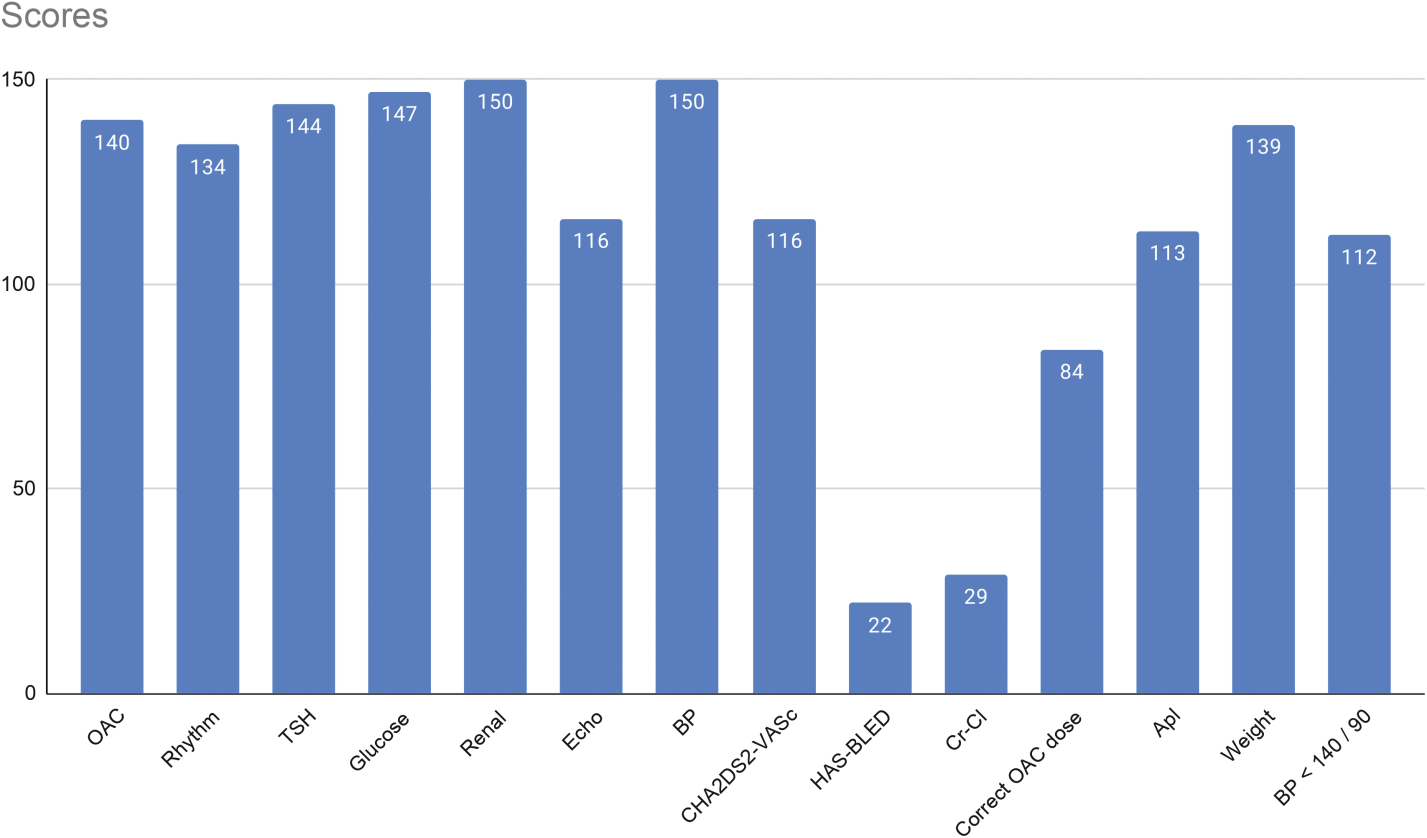
MAGIC Score.

Using the MAGIC score, no patient met all 14 guideline-based recommendation. Only 13.8% met 13 based recommendations. As illustrated on the graph, there is a sharp decline after a score of 10. The mean score was 11.3 points (Fig. 2). Points were most commonly deducted because the creatinine clearance and HAS-BLED score were not recorded, and the patient was not on the correct dose of OAC (Fig. 3).

Only 29 patients had their creatinine clearance calculated despite nearly all patients having their renal function checked (150 patients) since starting on OAC. Fifty-two patients were either taking Rivaroxaban, Edoxaban, or Dabigatran, which necessitates this calculation to ensure correct dosing.

## Discussion

### Key results

This study demonstrates the feasibility of using a quality of care score as an assessment tool for guideline adherence. In this cohort, the MAGIC score has shown that the majority of quality indicators described are being achieved.

The areas of the MAGIC score that had the poorest results were the recording of a HAS-BLED score and the recording of creatinine clearance, which are essential components of OAC management. These are amenable to electronic recording and calculation.

Twenty-two patients in this study had a HAS-BLED score recorded. Carrying out an individual stroke and bleeding risk assessment is a key component of AF management. Using the HAS-BLED score has been shown to improve appropriate OAC prescribing.^26^ In the mAFA-II trial, using the HAS-BLED score to dynamically assess bleeding risk was associated with increased prescribing of OAC, reduced bleeding events, and mitigation of modifiable risk factors for bleeding.^16^ Poor compliance with the assessment of bleeding risk means that those at higher risk of bleeding are not identified and, therefore, not managed correctly.

Most patients (86%) in this pilot study were on OAC. Based on the patient’s CHA_2_DS_2_-VASc score of 1 in a male or 2 in a female, OAC was indicated in 17 of the 21 patients not prescribed OAC. Of those taking NOACs, 80% were on the correct NOAC dose. Correct NOAC dosing requires an assessment of renal function. The dose reduction criteria for Apixaban include age, weight, and serum creatinine, whereas reduction of Rivaroxaban, Edoxaban, and Dabigatran is based on creatinine clearance. In this pilot study, 20% of patients (*N* = 21) taking a NOAC were on an incorrect dose. Inappropriate dosing can lead to significant adverse events such as bleeding, thromboembolic events, and increased all-cause mortality.**^18,27^** Calculation of creatine clearance using the Cockcroft-Gault equation takes age, weight, sex, and creatinine into account and could be automated on the general practice software system.

In primary care, the focus is on rate control in those with permanent AF. Patients in this study had a mean heart rate of 74 bpm, and only one patient with a heart rate of >110 bpm was identified. Beta blockers with the addition of Digoxin were the most common rate controlling medications used (Fig. 1). AADs were potentially inappropriately prescribed in 18 patients. There is no role for long-term AAD in those with permanent AF (i.e. where no attempt to restore sinus rhythm is planned)^2^, and these patients may have been exposed to the side effect profile of these drugs unnecessarily.

Managing comorbidities is an important component of atrial fibrillation care. AF often results from a combination of underlying interacting factors leading to atrial remodelling/cardiomyopathy, including cardiovascular disease, cardiovascular risk-factor burden, co morbidities, and an unhealthy lifestyle.^1^ Hypertension is the most common aetiological factor for AF. The mean BP in in this cohort was 132/77 mmHG with 24.8% patients having a BP > 140/90 mmHg. The mean BMI was 29.2 kg/m^2,^ which is classed as overweight and nearly obese. Obesity not only increases the risk of AF but also the risk of ischaemic stroke and death.^1^ Weight loss has been shown to be associated with better atrial fibrillation control.^21^

Our data suggest that the care in Irish general practice is of a similar standard to other published work in this area. For example, Wijtvliet et al. conducted a randomized control trial comparing nurse-led care to usual care in those with atrial fibrillation.^28^ As a secondary end point they measured adherence to 7 guideline-based recommendations; appropriate anticoagulation, thyroid function testing, echocardiography, appropriate application of rhythm control recommendations, renal function assessment, glucose level assessment, and blood pressure measurement.^28^ These 7 guideline-based recommendations are similar to those included in the MAGIC score. Wijtvliet et al. found that compliance to these recommendations was 61% under specialist nurse-led care and 26% in usual care.^28^ We found that 59.5% of patients met these same 7 guideline-based recommendation in the general practice cohort that we assessed.

Most patients in our study had a CDM review involving attendance with a general practice nurse and GP review at 6 monthly intervals. International guidelines for clinical AF management have recommended integrated care as the leading approach to manage AF resulting in improved clinical outcomes.^1,9–11,29^ Given that part of integrated care is the adherence to guideline-directed therapy,^12^ we hypothesize that the structured CDM programme in Ireland contributes to the high level of guideline adherence.

Previous studies have shown the importance of structured care in general practice.^9,29,30^ For example, a large RCT by van den Dries et al. demonstrated a significant reduction in all-cause mortality through an intervention involving regular review by nurses, monitoring of anticoagulation and easy access to specialist advice.^29^ This displays the value of integrated care, and a score such as MAGIC could be useful to help identify patients who need intervention. A further study of the effect of this approach is required.

A number of interventions have been tested, which have been shown to improve quality of care in chronic diseases. The American Heart Association’s (AHA) ‘Get With The Guidelines’ (GWTG) programme has been shown to improve the quality of care across a number of different cardiovascular diseases and has now been expanded to AF.^13^ Participating hospitals record patient details on a quality improvement registry and guideline adherence is facilitated using rapid – cycle quality improvement strategies and site-specific reporting.^13^ Sites are provided with performance data every 3 months which enables targeted interventions to be utilized mainly through education and support.^13^ The approaches used includes educational intervention, availability of treatment algorithms, and decision support tools.^31^ Using the GWTG approach, appropriate OAC prescription in patients with AF improved significantly demonstrating that a high level of guideline adherence is possible.^31^ The IMPACT-AF trial had similar findings. By using a multifaceted and multilevel educational intervention, the appropriate prescription for OAC significantly increased.^32^

These QI interventions have also been used successfully in primary care. It has been shown that a combination of education and specialist support resulted in an improvement in OAC prescribing and the number of patients undergoing both stroke and bleeding risk assessments.^30^

A group of general practitioners in the UK have developed an interactive dashboard to provide feedback on quality in AF care. They found that the dashboards were an acceptable and feasible way to communicate a summary of AF care.^33^ The prescribing section was rated as one of the most useful by the pilot group of GPs. Translating these approaches to the range of quality indicators in the MAGIC score could prove useful in improving the management of atrial fibrillation in general practice.

### Strengths and limitations

The MAGIC score enables the identification of gaps in AF care and guideline adherence. This score is feasible and could be replicated across other general practice sites as the information we collected is readily available within the electronic health record. This data set was obtained using proportionate sampling across 11 practices providing a representative sample of care in Ireland. This type of score readily identifies areas that can be targeted with an intervention to improve the quality of care and is based on internationally recognized quality of care markers.

A number of limitations apply to this study also. We recognize that the threshold for OAC varies according to guideline and geography. The European Society of Cardiology recommends offering OAC to those with ≥1 non-sex stroke risk factors.^1^ The EHRA QIs are derived from these guidelines, and therefore appropriateness of OAC was defined as CHA_2_DS_2_-VASc score of ≥1 for men and ≥ 2 for women in the QIs and in this pilot study.^1,2^

In contrast, the 2014 American College of Cardiology/American Heart Association guidelines and the 2019 focussed update recommend OAC with a CHA_2_DS_2_-VASc score of ≥ 2 for men and ≥ 3 for women.^34,35^ Similarly, the NICE guidelines recommend OAC for those with a score of 2 or more on the CHA_2_DS_2_-VASc score.^36^

Even at higher CHA_2_DS_2_-VASc scores, OAC may not be appropriate due to patient-specific factors. In this study, for those not on anticoagulation or incorrect doses according to guidelines, we did not ascertain if there were reasons for the non-use or use of different NOAC doses. We also did not record the time in target range (TTR) for patients on warfarin.

We assessed if patients had ever had the relevant investigations since diagnosis of AF but did not record the time since diagnosis to the performance of the relevant investigation.

This MAGIC score has not been validated, and we do not know if a higher score translates to better clinical outcomes, although other work in this area has demonstrated that adherence to guidelines is beneficial for patients.^4,5,29^ Overall, this was a pilot feasibility study to develop and pilot the MAGIC score and these outcome measures were outside the scope of the project. In order to validate the MAGIC score, we intend to conduct a randomized control trial using a mixed methods approach to evaluate if patients with a higher MAGIC score have better outcomes.

## Conclusion

The ESC highlights the importance of measuring quality with quality/performance indicators to identify opportunities for improvement.^1^ We propose that the MAGIC score can be used for this purpose. In this pilot study, the quality of AF care in Irish general practice is good and comparable with other published work. The MAGIC score has highlighted some key areas that can be targeted with intervention to improve the quality of care. Decision support tools, educational interventions, and visual summaries such as dashboards have all been shown to be effective QI strategies to improve guidelines adherence and will help inform future work.

## Supplementary Material

cmae001_suppl_Supplementary_Checklist

## Data Availability

The data underlying this article will be shared on reasonable request to the corresponding author.
